# Diagnostic performance of instantaneous wave-free ratio for the evaluation of coronary stenosis severity confirmed by fractional flow reserve: A PRISMA-compliant meta-analysis of randomized studies

**DOI:** 10.1097/MD.0000000000004774

**Published:** 2016-09-09

**Authors:** Wanrong Man, Jianqiang Hu, Zhijing Zhao, Mingming Zhang, Tingting Wang, Jie Lin, Yu Duan, Ling Wang, Haichang Wang, Dongdong Sun, Yan Li

**Affiliations:** a Department of Cardiology, Xijing Hospital; b Department of Health Statistics, Faculty of Preventive Medicine; c Department of Cardiology, Tangdu Hospital, Fourth Military Medical University, Xi’an, Shaanxi, China.

**Keywords:** fractional flow reserve (FFR), instantaneous wave-free ratio (iFR), ischemia, meta-analysis

## Abstract

**Background:**

The instantaneous wave-free ratio (iFR) is a new vasodilator-free index of coronary stenosis severity. The aim of this meta-analysis is to assess the diagnostic performance of iFR for the evaluation of coronary stenosis severity with fractional flow reserve as standard reference.

**Methods:**

We searched PubMed, EMBASE, CENTRAL, ProQuest, Web of Science, and International Clinical Trials Registry Platform (ICTRP) for publications concerning the diagnostic value of iFR. We used a random-effects covariate to synthesize the available data of sensitivity, specificity, positive likelihood ratio (LR+), negative likelihood ratio (LR−), and diagnostic odds ratio (DOR). Overall test performance was summarized by the summary receiver operating characteristic curve (sROC) and the area under the curve (AUC).

**Results:**

Eight studies with 1611 subjects were included in the meta-analysis. The pooled sensitivity, specificity, LR+, LR−, and DOR for iFR were respectively 73.3% (70.1–76.2%), 86.4% (84.3–88.3%), 5.71 (4.43–7.37), 0.29 (0.22–0.38), and 20.54 (16.11–26.20). The area under the summary receiver operating characteristic curves for iFR was 0.8786. No publication bias was identified.

**Conclusion:**

The available evidence suggests that iFR may be a new, simple, and promising technology for coronary stenosis physiological assessment.

## 1 Introduction

Fractional flow reserve (FFR) is an important physiological measurement that is used as the reference standard for assessing the functional significance of coronary artery stenosis in the catheter laboratory.^[1]^ The FFR-guided revascularization strategy is classified as a Class IA recommendation in the 2014 ESC/EACTS guidelines on myocardial revascularization.^[2]^ However, FFR is an index that requires an invasive procedure, expensive devices, and pharmacological intervention to induce maximal hyperemia. In clinical practice this is most commonly realized through using vasodilator intervention. Despite evidence demonstrating the efficacy of FFR-guided percutaneous coronary intervention (PCI), it has not been widely adopted because of the expense and lurking side effects caused by vasodilator administration.^[3–6]^

The instantaneous wave-free ratio (iFR) is a new vasodilator-free index of coronary stenosis severity, calculated as a trans-lesion pressure ratio during a specific period of baseline diastole, when distal resistance is the lowest and stable.^[7]^ The feasibility and diagnostic performance of iFR were reported in several studies.^[7–10]^ This method contributes to a wise therapeutic decision of interventional cardiologists and has the potential of being a reliable gatekeeper to PCI. The relationship between iFR and FFR has also been verified in a series of studies.^[11,12]^

Based on meta-analysis, the paper aims to summarize the latest high-quality literatures on the diagnostic performance of iFR, which assesses the functional significance of coronary stenosis.

## 2 Methods

We conducted this meta-analysis following Preferred Reporting Items for Systematic Reviews and Meta-Analyses (PRISMA) statement.

### 2.1 Ethical statement

The meta-analysis is based on the review of previous published articles. No ethical approval and patient consent were necessary.

### 2.2 Literature search

PubMed, EMBASE, Cochrane Central Register of Controlled Trials (CENTRAL), ProQuest Central, Web of Science, and International Clinical Trials Registry Platform (ICTRP) were searched in October 2015 to identify eligible diagnostic test accuracy (DTA) trials evaluating the diagnostic performance of iFR in patients with FFR as the reference. The keywords were “instantaneous wave-free ratio” or “iFR” and “fractional flow reserve” or “FFR,” with no other filter. No language restrictions were applied. Reference lists of retrieved records and relevant reviews were also screened. Ongoing studies were identified from ClinicalTrials.gov (http://clinicaltrials.gov/).

### 2.3 Inclusion and exclusion criteria

Inclusion criteria were as follows: the design was a diagnostic accuracy study; the participants were adult (18 years and older) patients with suspected or known coronary artery disease (CAD); the index test was iFR; the reference standard test was FFR; the data of true positive (TP), false positive (FP), true negative (TN), false negative (FN), sensitivity and specificity could be retrieved from the published full text.

Studies were excluded by the following criteria: studies were not conducted humans (studies on animals or in vitro systems); the literature did not report diagnostic performance results of iFR; the literature was prognostic studies, reviews, case reports, and comments; there was possible overlapping of study samples or overt verification bias.

Two investigators (WM, JH) selected studies independently and disagreements were resolved by discussion among all authors.

### 2.4 Data extraction and quality assessment

The following data of eligible studies were documented: the name of the study, the first author, the year of publication, and details of the study design; characteristics of patients; iFR and FFR parameters; the data of TP, TN, FP, FN, sensitivity and specificity presented in a 2 × 2 table; the area under the curve of receiver operating characteristics (AUC).

The Review Manager 5.3 (Cochrane collaboration) with Quality Assessment of Diagnostic Accuracy Studies 2 (QUADAS-2) was applied to evaluate the study quality. Data extraction and quality assessment were performed by 3 investigators (MZ, TW, DS) independently and disagreements were resolved by discussion among all authors.

### 2.5 Data synthesis and analysis

The sample size of each study dominates the weighted average of the meta-analysis. Pooled sensitivity, specificity, positive likelihood ratio (LR+), negative likelihood ratio (LR−), and diagnostic odds ratio (DOR) were calculated using suitable model which was decided by heterogeneity evaluation. On the basis of the random-effects analysis, heterogeneity was calculated by Chi-square and Cochran *Q* test. For *I*^2^, values between 25% and 50% were considered low, between 50% and 75% were considered medium, and above 75% were considered high.^[13]^

Diagnostic threshold variation among publications was evaluated by calculating Spearman correlation coefficient and constructing summary receiver operating characteristic (sROC) curve, which were estimated from D = a + bS, where D is the difference of the logits (log odds) of the true positives (sensitivity) and false positives (1 − specificity) and S is the sum of these logits. The area under the curve of receiver operating characteristics (AUC) was explored. *Q* point in sROC is the point where the curve is intersected by a diagonal running from the top left to the right bottom corner of the sROC space and provides an estimate of composite sensitivity and specificity.

The sROC was used to determine the DTA. Pragmatically, AUC between 0.75 and 0.92 represented a high degree of diagnostic accuracy, and AUC between 0.93 and 0.96 was considered more accurate.^[14,15]^

Publication bias was assessed visually by a scatter plot of the diagnostic log odds ratio (lnDOR) versus the inverse of the square root of the effective sample size (1/root (ESS)), which exhibits a symmetrical funnel shape when publication bias is absent. The *P*-value of less than 0.05 for the slope coefficient indicated significant asymmetry.^[16]^

Meta-regression analysis was performed to identify sources of heterogeneity. Predefined sources of heterogeneity included study design (iFR cut-off), patient characteristics (mean age, proportion of men), proportion of medical history (diabetic patients, hypertension, and current smoker).

The meta-analysis was performed by RevMan 5.3 (Cochrane collaboration), STATA 12.0 and MetaDiSc (Version 1.4, Clinical Biostatistics Unit, Hospital Ramony Cajal, Madrid, Spain).

## 3 Results

### 3.1 Study selection and characteristics

**Figure 1:**
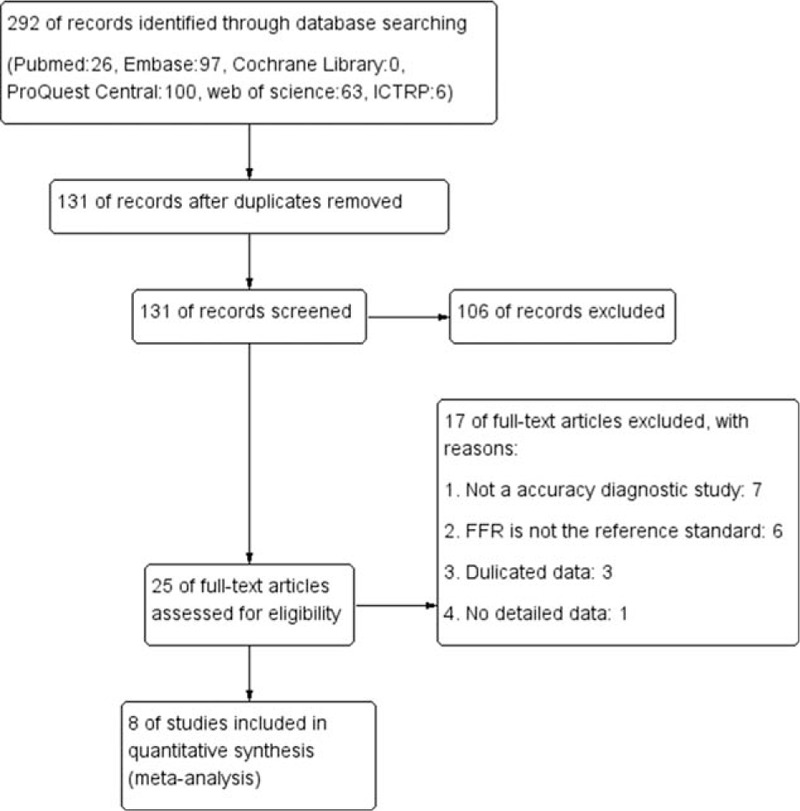
Flow diagram of search and study selection.

The initial search obtained 292 potentially relevant publications. After screening by title and abstract, 25 full articles were retrieved. Eight studies were finally included in this study after the abstracts and full texts were reviewed.^[7,10,17–22]^ The flow chart of articles research and selection process are demonstrated in Fig. 1.

These studies enrolled 1611 patients and 2029 lesions. Baseline characteristics of these studies and subjects are shown in Tables 1 and 2. Diagnostic characteristics of these studies are shown in Table 3.

**Table 1:**
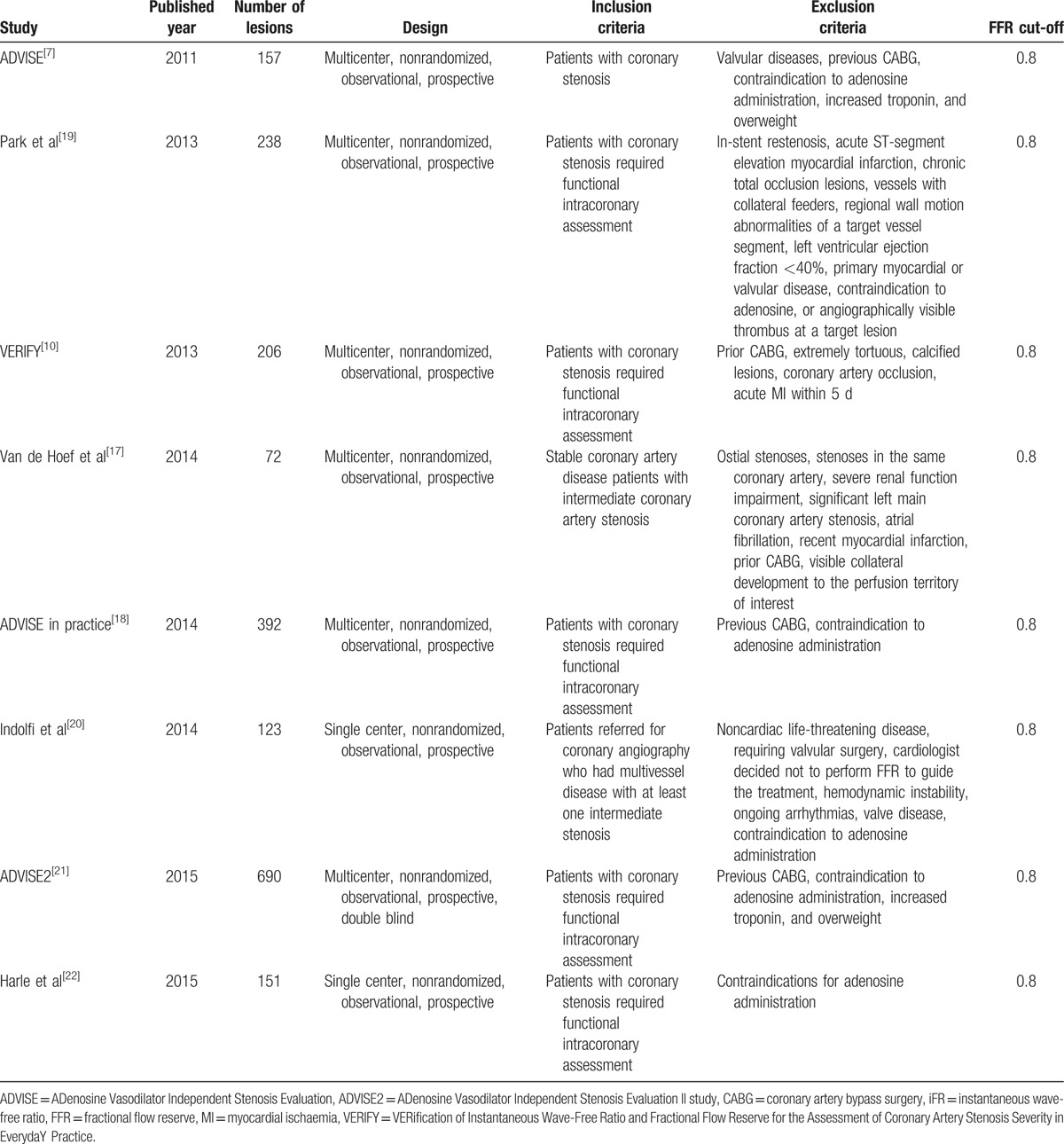
Summary characteristics of included studies.

**Table 2:**
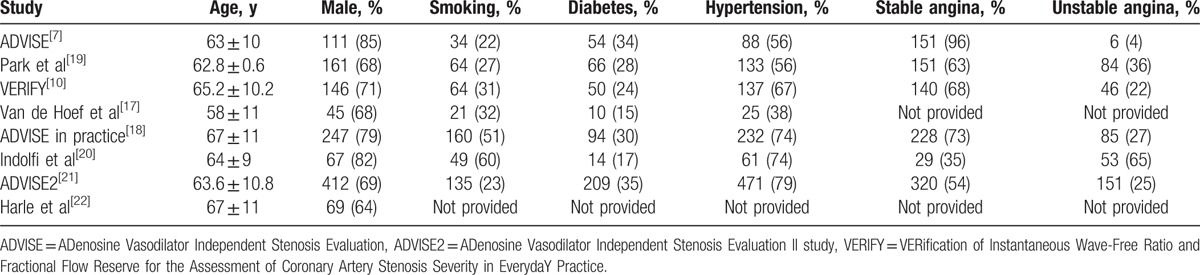
Baseline data and cardiovascular risk factors of study population.

**Table 3:**
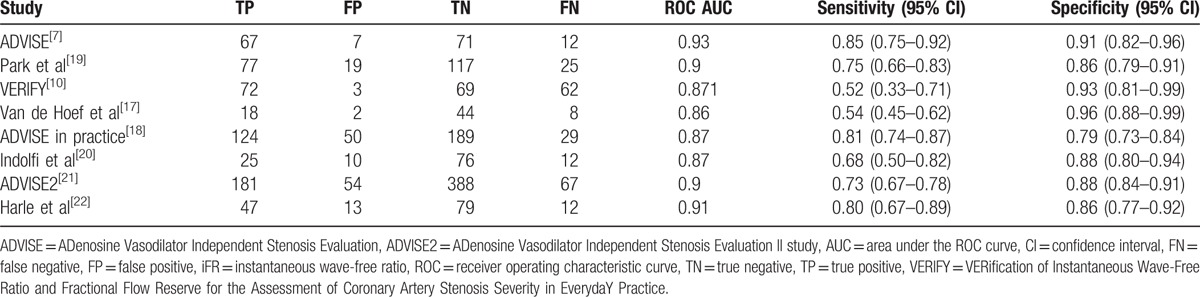
Individual study estimates of diagnostic accuracy of iFR for detection of ischemia-causing lesions.

**Figure 2:**
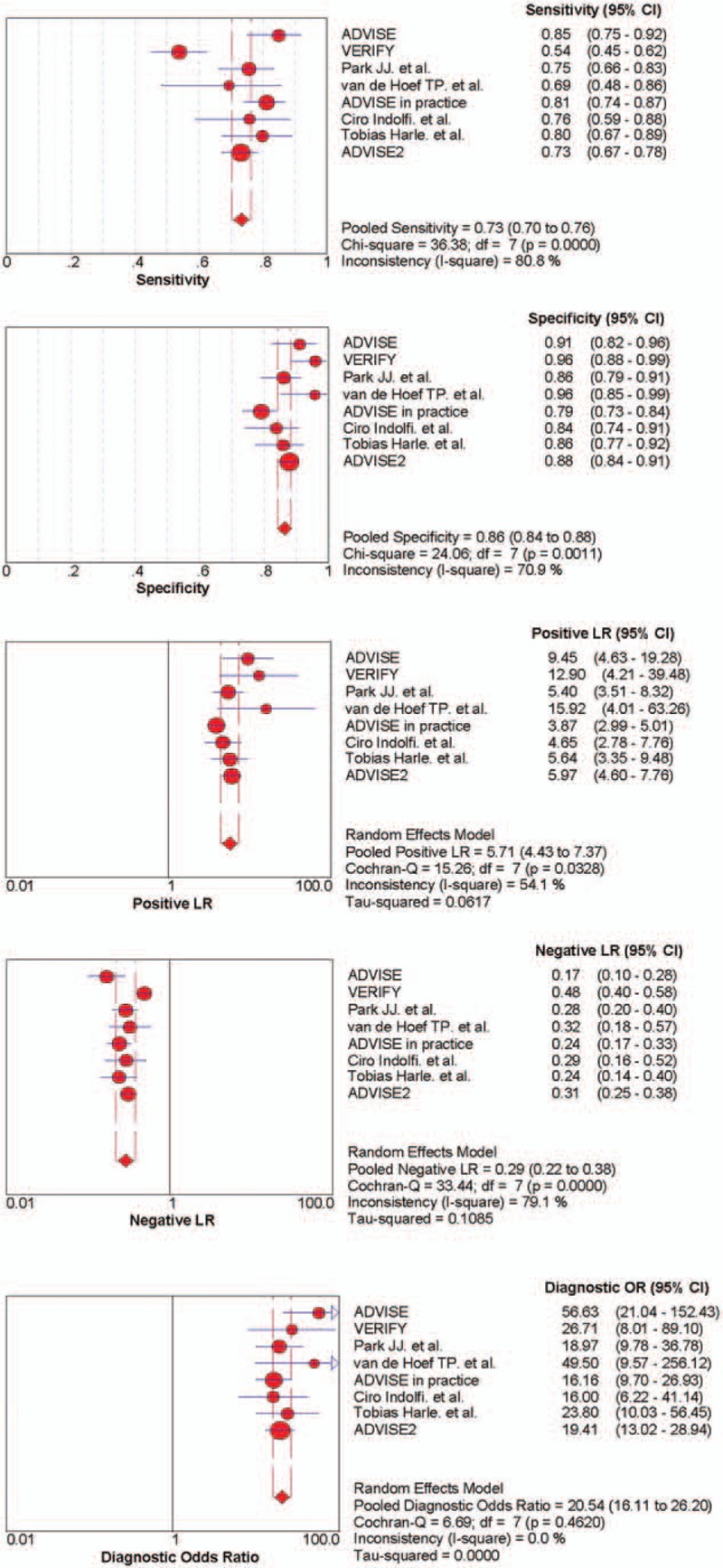
Risk of bias and applicability summary.

The overall quality of the studies according to the QUADAS-2 tool was high. The methodological quality of individual studies is presented in Fig. 2.

### 3.2 Diagnostic accuracy of iFR

**Figure 3:**
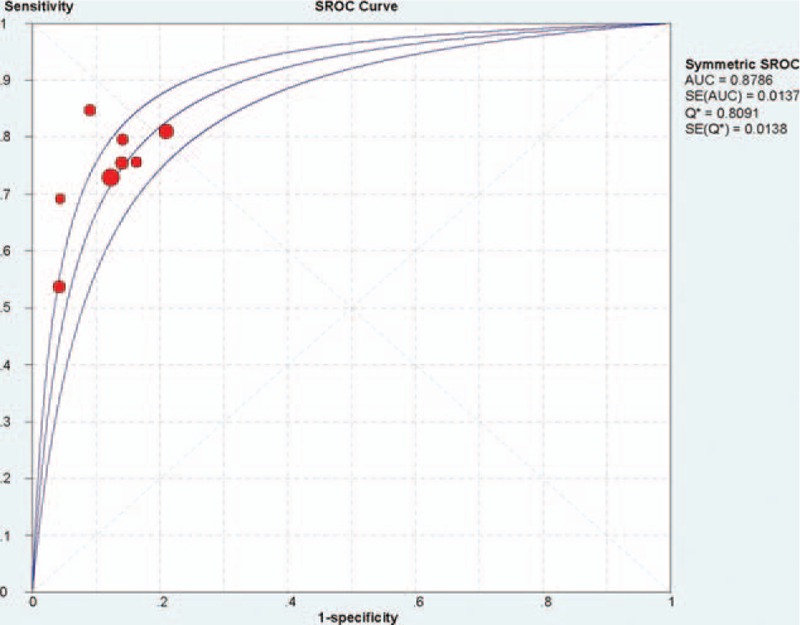
Forest plots for sensitivity, specificity, LR+, LR−, and DOR of iFR for the detection of coronary ischemia causing stenosis.

A total of 1611 patients and 2029 lesions were analyzed. The pooled estimate of sensitivity for the detection of lesion-specific ischemia at the per-lesions level was 73.3% (95% CI, 70.1–76.2%) using a fixed-effects model. The corresponding pooled estimate of specificity was 86.4% (95% CI, 84.3–88.3%) using a fixed-effects model. Heterogeneity was found for both sensitivity (*I*^2^ = 80.8%, *P* < 0.001) and specificity (*I*^2^ = 70.9%, *P* < 0.001). The pooled estimate of LR+ and LR− were 5.71 (95% CI, 4.43–7.37) and 0.29 (95% CI, 0.22–0.38). The pooled estimate of DOR was 20.54 (95% CI, 16.11–26.20) (Fig. 3).

### 3.3 Diagnostic threshold effect

For iFR, spearman correlation coefficients were 0.619 (*P* = 0.102), indicating that the diagnostic threshold effect might exist in iFR data. Therefore, symmetrical sROC curve was drawn.

**Figure 4:**
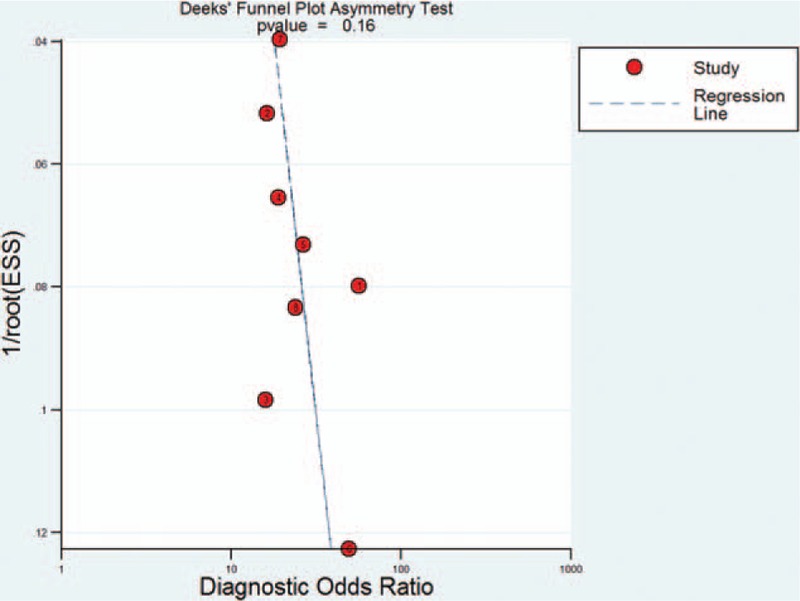
Summary receiver operating characteristic (sROC) curve for iFR using random-effects model.

A random-effects sROC model was employed to fit a single symmetric sROC curve because of the high degree of heterogeneity. The AUC for iFR was 0.8786 (Fig. 4).

### 3.4 Meta-regression analysis

The meta-regression analysis, using the predefined potential sources of heterogeneity as covariates in the Moses–Shapiro–Littenberg model, indicated that the iFR cut-off value (*P* < 0.05) might be significant predictors. That means the diagnostic threshold effect existed in iFR data. However, other factors did not influence the diagnostic accuracy, including age (*P* = 0.72), sex (*P* = 0.48), the prevalence of diabetes (*P* = 0.57), hypertension (*P* = 0.48), and smoking habit (*P* = 0.18).

### 3.5 Publication bias

**Figure 5:**
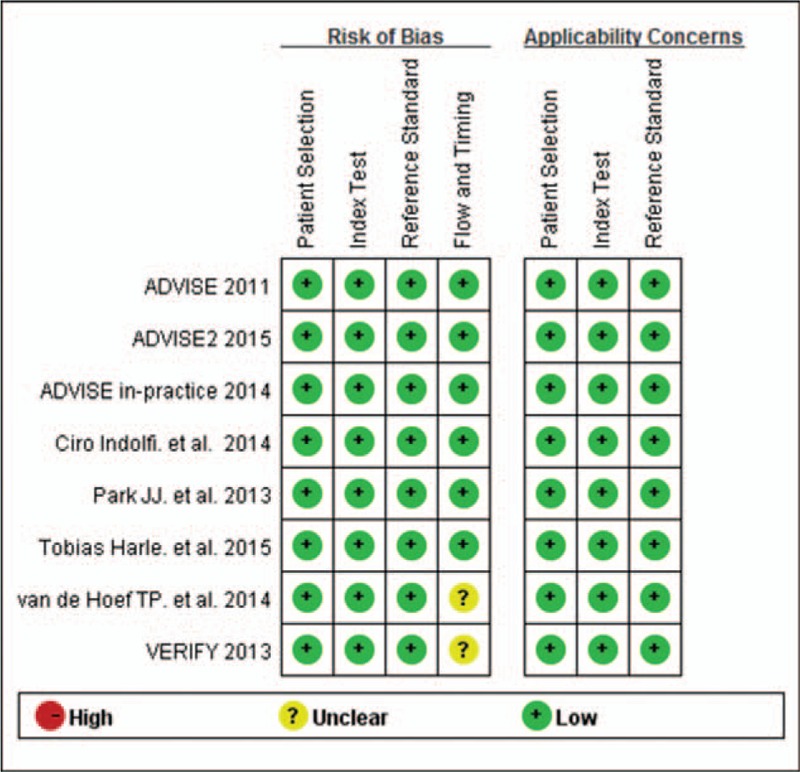
Deeks funnel plot for studies.

The publication bias was assessed by Deeks funnel plot asymmetry test. The plot resembled a symmetrical funnel shape, and the *P*-value for the Deeks funnel plot asymmetry test was 0.16. Therefore, publication bias was unlikely (Fig. 5).

## 4 Discussion

Coronary physiology has gained much attention in the light of guideline recognition, ongoing clinical research, and new technologies.^[2,23,24]^ Determining the physiological importance of a stenosis during angiography is pivotal in improving the prognosis of patients with CAD. Accumulating evidences have demonstrated that vascular revascularization strategies targeting to ischemia-causing stenosis maximizes the benefit of PCI and coronary artery bypass grafting (CABG) while minimizing the risk.^[25,26]^

FFR-guided PCI has been demonstrated to be superior to angiography-guided PCI. Significant improvement in clinical outcomes and quality of life were observed.^[5,27,28]^ Although accumulating evidence have indicated that FFR-guided therapy was inversely correlated with incidence of major adverse cardiac events (MACE). Some limitations still lies in the application of FFR-guided therapy. First of all, when using different types of hyperemic agents such as adenosine or nicorandil, the hemodynamic reaction may be different which will affect the results of FFR.^[29]^ Secondly, in response to hyperemic agents, some patients may develop paradoxical vasoconstriction of the microcirculatory bed which may also affect the FFR value. Thirdly, patients with microvascular dysfunction usually have reduced hyperemic flow levels and increased FFR value, which may cause the bias when interpretating the FFR results.^[30]^

Interestingly, Tarkin and colleagues observed 7 different hemodynamic responses after adenosine infusion. They found that during peak and stable hyperemia stages, the change behaviors of proximal (Pa) and distal (Pd) pressure were completely different. Different reactions to adenosine infusion will change the ratios of Pd/Pa, thus change FFR value during peak and stable hyperemia stages.^[31]^ These findings suggest that pressure-based FFR and true flow-based FFR may not exactly the same which may affect by hemodynamic changes.

iFR is a resting index of stenosis severity that provides a physiological quantification of the effect of a stenosis on the coronary circulation. iFR is measured during a specific period of diastole known as the wave-free period, when flow is intrinsically at its highest compared with the whole cycle. Thus, iFR allows physiological assessment of coronary stenosis under rest conditions free of the side effects of hyperemic agent.^[7]^

The results of this meta-analysis indicated that iFR as a resting index of stenosis severity presented the modestly effective pooled sensitivity, specificity, and AUC. The numerical values were 73.3% (sensitivity), 86.4% (specificity), and 0.8786 (AUC). The results were similar to other research.^[12]^ Although iFR are imperfect surrogates of FFR close to the clinically used cut-off value of 0.80, they may still provide acceptable accuracy at greater or lesser degrees of functional stenosis severity.^[8]^ As with any diagnostic test FFR, iFR have inherent variability. On the basis of the present report and consistent with prior studies, the universal adoption of iFR may not be recommended.^[9,10]^

The fundamental basis of iFR approximation to FFR is the assumption that diastolic resting myocardial resistance equals mean hyperemic resistance.^[7]^ However, the actual diastolic resting myocardial resistance does not equal to mean hyperemic resistance. This may cause a difference decision according to the iFR value as compared to the FFR value.^[7,9]^ The results of iFR and FFR are mostly consistent. However, we should keep in mind that in some individual cases, iFR may provide an uncertain estimate of FFR.

To overcome these limitations, a hybrid iFR–FFR approach has been proposed as a way to translate into practice the potential value of iFR as a diagnostic tool. The ADVISE II (ADenosine Vasodilator Independent Stenosis Evaluation II) study supports the diagnostic value of this hybrid iFR–FFR diagnostic approach. With this strategy, adenosine would not be required in 69% of the stenoses, and in 65% of patients, adenosine would not be needed at all. These figures support the potential of iFR to ease catheterization laboratory workflow and to reduce costs associated with ischemia-driven revascularization.^[21]^

Further to using FFR as a reference technique to assess iFR, future studies must focus on demonstrating noninferiority of iFR with respect to FFR in terms of clinical outcomes when it is used as a decision-making tool. DEFINE-FLAIR (NCT02053038) is a multicenter, double-blind, randomized controlled trials, which will be the largest physiology study performed to date enrolling 2500 patients with intermediate coronary artery stenosis in both stable patients and those with acute coronary syndrome (ACS). Patients will be randomized to either an iFR-guided approach (treatment threshold iFR < 0.90) or an FFR-guided approach (treatment threshold FFR ≤ 0.80). The study is powered to test Major Adverse Cardiovascular Events (MACE) at 1 year for noninferiority between iFR and FFR.^[22]^ The iFR-SWEDEHEART (NCT 02166736) study is simultaneously being performed in Sweden in which 2000 patients will be randomized to either iFR or FFR-guided group. Such trials may provide more information about the feasibility of iFR-guided revascularization.^[32]^

The present meta-analysis shares the limitations of its primary sources. First, the number of included studies in the meta-analysis was relatively small. Second, we failed to identify all the sources of heterogeneity among the included studies using performing meta-regression because of the limited data reported. Third, the validity of our results is dependent on the validity of the studies included. Finally, the induction of ischemia in each study was not consistent which may bias the results.

## 5 Conclusions

With FFR as the reference standard, the diagnostic ability of iFR to detect coronary stenosis severity is high. iFR may be a new, simple, and promising technology for coronary stenosis physiological assessment.

## Acknowledgments

The Dr. Wanrong Man acknowledges his friend, Mr. Hailong Wu, for his encouragement (Master Candidate, Baker Program in Real Estate in Cornell University, Queens, NY). Mr. Wu declares no competing interests.
